# Korrelation zwischen Komorbiditäten und Veränderungen des Lungenparenchyms im CT des Thorax bei Patienten mit COVID-19-Pneumonie

**DOI:** 10.1007/s00063-023-01062-3

**Published:** 2023-09-25

**Authors:** Nima Nadem Boueini, Patrick Haage, Nadine Abanador-Kamper, Lars Kamper

**Affiliations:** 1 Private Universität Witten/Herdecke, Alfred-Herrhausen-Straße 50, 58455 Witten, Deutschland; 2 Diagnostische und interventionelle Radiologie, HELIOS Universitätsklinikum Wuppertal, Heusnerstr. 40, 42283 Wuppertal, Deutschland; 3 Abteilung für Kardiologie, HELIOS Klinikum Elberfeld, Arrenberger Str. 20, 42117 Wuppertal, Deutschland

**Keywords:** COVID-19-Pandemie, Pulmonale Bildgebung, Radiologie, Pneumologie, Risikofaktoren, COVID-19 pandemic, Lung imaging, Radiology, Pneumology, Risk factors

## Abstract

**Hintergrund und Ziel der Arbeit:**

Die pulmonale Manifestation von COVID-19 wird anhand standardisierter CT-morphologischer Kriterien beschrieben. In dieser Studie untersuchten wir mögliche Assoziationen zwischen dem CT-morphologischen Infiltratmuster bei COVID-19-Pneumonie und typischen Komorbiditäten sowie dem klinischen Verlauf.

**Methoden:**

Wir analysierten klinische Daten und pulmonale Bildgebung von 61 Patienten mit positivem PCR-Test. Die pulmonalen Veränderungen wurden kategorisiert und auf Zusammenhänge mit vorbestehenden Komorbiditäten und dem klinischen Verlauf überprüft.

**Ergebnisse:**

Im Vergleich zu Patienten mit untypischen Infiltratmustern (2/19, 10,5 %) wurden 25 Patienten mit typischen Infiltratmustern (25/42, 59,5 %) signifikant häufiger intensivmedizinisch behandelt (*p* < 0,001). Außerdem erhielten Patienten mit typischen Infiltratmustern im Vergleich zu Patienten mit untypischen Infiltratmustern häufiger eine nichtinvasive Beatmung (12/42, 28,6 %, *p* = ,040) und High-flow-Therapie (8/42, 19 %, *p* = 0,041). Die Mortalität war ebenfalls höher bei Patienten mit typischen Infiltratmustern, wobei 15 Patienten (15/42, 35,7 %) im Verlauf verstarben verglichen mit nur einem Patienten mit untypischem Infiltratmuster (1/19, 10,5 %, *p* = 0,012). Es konnte kein signifikanter Zusammenhang zwischen spezifischen Komorbiditäten und dem resultierenden Infiltratmuster nachgewiesen werden.

**Diskussion:**

Patienten mit einem typischen COVID-19-Infiltratmuster werden häufiger intensivmedizinisch behandelt und weisen eine höhere Mortalität auf. Weitere Analysen mit größeren Patientenkollektiven sind notwendig, um spezifische Risikofaktoren für eine typische COVID-19-Pneumonie zu identifizieren.

## Abstract (en)

**Background and objectives:**

Pulmonary manifestation of coronavirus disease 2019 (COVID-19) is described using standardized computed tomography (CT) morphologic criteria. In this study, we investigated possible associations between thoracic CT manifestations in COVID-19 pneumonia and typical comorbidities, as well as clinical course.

**Methods:**

We analyzed clinical data and pulmonary imaging of 61 patients with positive PCR test. Pulmonary changes were categorized and reviewed for associations with pre-existing comorbidities and clinical course.

**Results:**

Compared to patients with atypical infiltrate patterns (2/19, 10.5%), 25 patients with typical infiltrate patterns (25/42, 59.5%) were significantly more likely to receive intensive care (*p*<0.001). In addition, patients with typical infiltrate patterns were more likely to receive non-invasive ventilation (12/42, 28.6%, *p*=0.040) and high-flow therapy (8/42, 19%, *p*=0.041) compared to patients with atypical infiltrate patterns. Mortality was also higher in patients with typical infiltrate patterns, with 15 patients (15/42, 35.7%) dying during follow-up compared to only 1 patient with atypical infiltrate pattern (1/19, 10.5%, *p*=0.012). No significant association between specific comorbidities and the resulting infiltrate pattern could be demonstrated.

**Conclusions:**

Patients with a typical COVID-19 infiltrate pattern are more likely to receive intensive care and show higher mortality rates. Further analysis with larger patient collectives is needed to identify specific risk factors for typical COVID-19 pneumonia.

## Einleitung

Die Computertomographie (CT) des Thorax kann pulmonale Manifestationen einer COVID-19-Pneumonie visualisieren. Für die Beschreibung der bildmorphologischen Veränderungen empfiehlt die Radiological Society of North America (RSNA) eine strukturierte Befundungsvorlage mit folgenden 4 Kategorien [1]: typisch (typ), atypisch (atyp), unklar/„indeterminate“ (ind) und keine Pneumonie/blande (neg). Dadurch kann die prognostische Wahrscheinlichkeit einer vorliegenden COVID-19-Pneumonie eruiert werden. Warum es bei einigen Patienten zu bestimmten Infiltratmustern kommt, ist bisweilen unklar. In der vorliegenden Studie wurde der Zusammenhang zwischen einer typischen Infiltratausprägung und einzelnen Komorbiditäten bei COVID-19-Patienten untersucht. Die Arbeitshypothese ist, dass ein signifikanter Zusammenhang zwischen einzelnen Komorbiditäten und dem typischen Infiltratmuster im CT-Thorax besteht. Bisher existieren keine Studien, die den Zusammenhang zwischen bildmorphologischen Eigenschaften und vorliegenden Risikofaktoren bei COVID-19 analysiert haben.

## Material und Methoden

### Studiendesign und Patientenkollektiv

Im Rahmen einer retrospektiven monozentrischen Studie wurden die Daten von insgesamt 61 Patientinnen und Patienten über einen Zeitraum von 5 Monaten evaluiert. Die untersuchten Patienten wurden entweder stationär oder ambulant behandelt. Zu den Einschlusskriterien gehörten ein positiver Real-time-polymerase-chain-reaction(RT-PCR)-Test und eine klinisch indizierte CT-Untersuchung des Thorax zu Beginn der Krankheitsphase. Die Indikation der CT-Untersuchung wurde zusammen mit den zuweisenden Fachabteilungen, überwiegend die pneumologische Abteilung, getroffen. Das Projekt wurde von der lokalen Ethikkommission geprüft und genehmigt (Aktenzeichen: 143/2020). Patientenbezogene Daten wurden anonymisiert ausgewertet.

### Datensammlung

Die Patientendaten und Komorbiditäten wurden mittels klinikinternem RIS-System (Centricity, GE Health-Systems; Chicago, USA), und Klinikinformationssystem (SAP, Walldorf, Deutschland) analysiert.

### Bildakquisition und CT-Parameter

Die Bildgebung erfolgte an 2 baugleichen klinischen CT-Geräten (Somatom Definition Flash, Siemens Medical Systems, Deutschland) in Inspirationsstellung, mit folgenden Scanparametern: Röhrenspannung: 100 KV, Kernel: B30f und B70f, Kollimationsdicke: 0,6–5 mm, Röhrenspannung: 368–760 mAs.

### Bildanalyse

**Figure 1:**
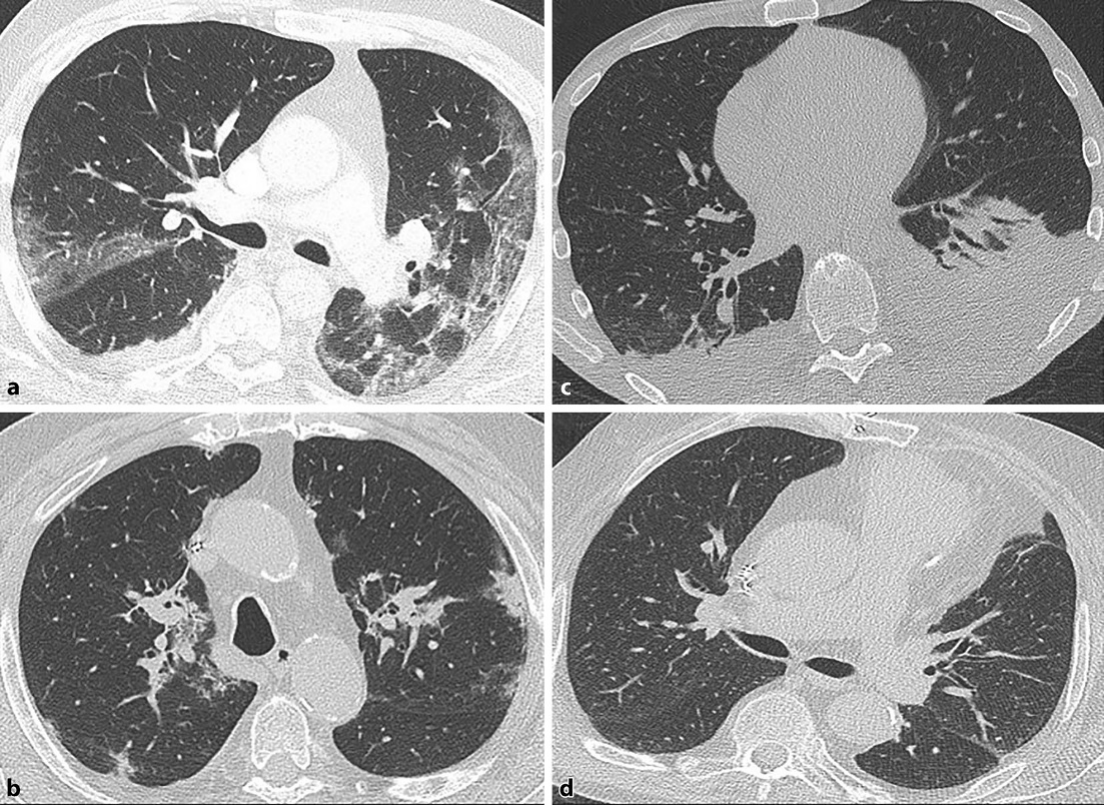

Die standardisierte Befundung der Thorax-CT erfolgte basierend auf der Befundvorlage der Radiological Society of North America (RSNA). Zu Beginn der Pandemie wurde das RSNA-Befundungsschema zeitnah eingeführt, um die Wahrscheinlichkeit einer vorliegenden COVID-19-Pneumonie zu eruieren, bevor das PCR-Ergebnis vorlag (Tab. 1). Dabei repräsentieren die Kategorien der RSNA-Befundvorlage kein Krankheitsstadium, sondern verschiedene pulmonale Ausprägungen einer COVID-19-Pneumonie (Abb. 1). Erfahrene Radiologen unserer Klinik befundeten die Untersuchungen und definierten im Befundtext die entsprechende Kategorie der RSNA-Befundvorlage. Die CT wurden von 10 Assistenzärztinnen und Assistenzärzten befundet und von 6 erfahrenen Oberärztinnen und Oberärzten validiert. Für die Studie wurden die Kategorien atypisch, unklar und blande zur Kategorie „untypisch“ (Gruppe UNTYP) zusammengefasst. Die Gruppe der Patienten mit typischen Veränderungen wurde hierbei belassen (Gruppe TYP). Siehe hierzu auch Unterkategorie „statistische Analyse“ im Methodenteil.

**Table 1:** 

Klassifikation	CT-Befund
Typisch	Periphere, bilaterale (multilobäre) Milchglastrübungen mit/ohne Konsolidierungen oder sichtbare intralobuläre Verdickungen („crazy paving“) Rundliche multifokale Milchglastrübungen mit/ohne Konsolidierungen oder intralobuläre Verdickungen („crazy paving“) „reverse halo sign“ (= umgekehrtes Halo-Zeichen = Milchglastrübung mitumgebender ringförmiger Konsolidierung) oder andere Zeichen einer organisierenden Pneumonie (können sich im späteren Krankheitsverlauf zeigen)
Unklar/„indeterminate“	*Fehlen typischer Zeichen einer COVID-19-Pneumonie und Vorhandensein von*: multifokalen, diffusen, perihilären oder unilateralen Milchglastrübungen mit/ohne Konsolidierungen, ohne typisches Verteilungsmuster und nichtrundlicher Form oder peripherer Verteilung einigen sehr kleinen Milchglastrübungen in nichtrundlicher Form oder peripherer Verteilung
Atypisch	*Fehlen typischer oder unklarer („indeterminate“) Zeichen einer COVID-19-Pneumonie und* *Vorhandensein von:* isolierten lobären oder segmentalen Konsolidierungen mit/ohne Milchglastrübungen diskreten Mikronoduli (zentrilobulär, „tree in bud“) Kavernenbildungen glatten interlobulären septalen Verdickungen mit Pleuraergüssen
Negativ/blande	Keine CT-morphologischen Zeichen einer Pneumonie

### Komorbiditäten und Untersuchungsparameter

Für die Studie untersuchten wir epidemiologische Parameter sowie folgende Komorbiditäten: arterielle Hypertonie (aHTN), koronare Herzkrankheit (KHK), Herzrhythmusstörungen (HRST), Diabetes mellitus Typ 2 (DM II), chronisch-obstruktive Lungenkrankheit (COPD), Asthma bronchiale, periphere arterielle Verschlusskrankheit (PAVK), stattgehabte Lungenarterienembolie (LAE), Adipositas (Übergewicht, Adipositas I–III), obstruktives Schlafapnoesyndrom (OSAS), Niereninsuffizienz sowie Refluxösophagitis.

Unter Niereninsuffizienz wurden akute und chronische Verläufe zusammengefasst. Zu den Herzrhythmusstörungen zählen in der vorliegenden Studie Vorhofflattern, Vorhofflimmern, AV-Blöcke und Tachykardien.

Patienten mit einer diagnostizierten COPD mit bekanntem GOLD-Stadium von mindestens 1 wurden aufgelistet.

Es wurden zudem bildmorphologische Parameter herangezogen und ebenfalls verglichen. Hierzu zählen Elemente wie Lappenbefall, Uni- oder Bilateralität, Intubation während der CT-Untersuchung, eine vorliegende LAE, Pleuraerguss und mediastinale Lymphadenopathie. Darüber hinaus wurde auch die Anzahl der CT-Untersuchungen im Lauf des Aufenthalts miterfasst.

Zusätzlich wurden die Intensivpflichtigkeit, die Mortalität und die Notwendigkeit einer extrakorporalen Membranoxygenierung (ECMO) als Outcomeparameter analysiert.

### Statistische Analyse

Die genannten Parameter wurden zunächst in einer Excel-Tabelle (Microsoft Office, Redmond, WA, USA) eingetragen. Die statistische Auswertung erfolgte nach Datenübertragung über die Software SPSS Vers. 28 (IBM, Chicago, IL, USA). Wir verglichen die Komorbiditäten der Patientinnen und Patienten mit dem Infiltratmuster der Thorax-CT nach dem RSNA-Schema und stellten die Häufigkeiten in einer deskriptiven Statistik dar. Hierbei wird ein typisches und untypisches Muster mit den Komorbiditäten verglichen, um einen möglichen Zusammenhang zu erkennen. Um die Übersichtlichkeit zu erhöhen und die statistische Auswertung zu erleichtern, wurde die Studienpopulation in 2 Gruppen aufgeteilt: TYP-Gruppe (für eine COVID 19-Pneumonie mit typischen Veränderungen) und die UNTYP-Gruppe (untypische Veränderungen für eine COVID-19-Pneumonie im Thorax-CT [RSNA-Kategorien: atypisch, unklar und blande]). Die Gruppe mit den untypischen Veränderungen (Gruppe UNTYP) darf allerdings nicht mit der RSNA-Kategorie „atypisch“ verwechselt werden. Anschließend wurden die 2 Gruppen hinsichtlich bildmorphologischer und Outcomeparametern analysiert. Als statistische Testverfahren wurde der χ^2^-Test genutzt. Ein *p*-Wert < 0,05 wurde als statistisch signifikant angesehen.

## Ergebnisse

### Demografische Parameter/Baselinecharakteristika

Die demografischen Patientendaten und vorbestehende Komorbiditäten sind in Tab. 2 aufgeführt.

**Table 2:** 

Baseline	*n*	%/MV ± SD
Männlich	36	59 %
Weiblich	25	41 %
Alter (Mittelwert)	–	68 ± 17
aHTN	37	60,7 %
KHK	15	24,6 %
HRST	6	9,8 %
COPD	9	14,8 %
Asthma	6	9,8 %
DM II	13	21,3 %
PAVK	3	4,9 %
Malignom	9	14,8 %
Zerebraler Insult	9	14,8 %
Übergewicht	14	23 %
Adipositas I	11	18 %
Adipositas II	2	3,3 %
Adipositas III	1	1,6 %
OSAS	5	8,2 %
NI	15	24,6 %
Reflux	8	13,1 %
Z. n. LAE	9	14,8 %

*aHTN* arterielle Hypertonie, *KHK* koronare Herzkrankheit, *HRST* Herzrhythmusstörungen, *COPD* chronisch obstruktive Lungenerkrankung, *DM II* Diabetes mellitus Typ II, *PAVK* periphere arterielle Verschlusskrankheit, *OSAS* Obstruktives Schlafapnoesyndrom, *NI* Niereninsuffizienz, *LAE* Lungenarterienembolie. *Z.* *n.* Zustand nach

In der vorliegenden Studienpopulation war das männliche Geschlecht etwas häufiger vertreten als das weibliche Geschlecht. Hierbei waren 36 Patienten männlich (59 %) und 25 weiblich (41 %). Das durchschnittliche Alter des Patientenkollektivs betrug 68 Jahre (±17). 37 Patienten (60,7 %) wiesen eine arterielle Hypertonie auf. 15 Patienten (24,6 %) litten unter einer KHK. 13 Patienten (21,3 %) hatten einen nachgewiesenen Diabetes mellitus vom Typ 2 (DM II). Unter einer Niereninsuffizienz litten 15 Patienten (24,6 %). 14 der untersuchten Patienten wiesen Übergewicht auf (23 %), 11 Patienten eine Adipositas 1. Grades (18 %), 2 Patienten eine Adipositas 2. Grades (3,3 %) und ein Patient eine Adipositas 3. Grades (1,6 %). Patienten (9,8 %) litten an einer Herzrhythmusstörung (HRST). Neun Patienten (14,8 %) wiesen eine COPD mit mind. GOLD Stadium I auf. Unter einer PAVK litten 3 Patienten (4,9 %). Neun Patienten hatten einen zerebralen Insult durchgemacht (14,8 %). Bei 5 Patienten (8,2 %) war ein obstruktives Schlafapnoesyndrom (OSAS) nachgewiesen.

Neun Patienten des Studienkollektivs hatten eine Vorgeschichte mit einer arteriellen Lungenembolie (14,8 %). Aufgrund des auffällig häufigen Auftretens einer Refluxkrankheit wurde diese mit in die Evaluation einbezogen. Insgesamt 8 Patienten des Patientenkollektivs hatten eine Refluxkrankheit (13,1 %).

### Bildmorphologische Parameter

Die Tab. 3 zeigt eine Auflistung der untersuchten bildmorphologischen Parameter. Die Infiltratmuster wurden, wie zuvor erwähnt, nach dem RSNA-Befundungsschema klassifiziert in typische (typ), atypische (atyp), „indeterminate“ (ind) und keine (neg) Pneumonie. Zur statistischen Analyse wurden die nichttypischen und fehlenden Befunde (atyp = 5, ind = 7 und neg = 7 Patienten) zusammengefasst und den typischen COVID-Infiltraten (typ = 42 Patienten) gegenübergestellt. Es resultierten 42 Patienten (68,9 %) mit typischem Infiltratmuster und insgesamt 19 Patienten (31,2 %) mit nichttypischen Befunden. Von den insgesamt 61 Patienten hatten 51 einen bilateralen Befall (83,6 %), 3 Patienten einen unilateralen Befall (4,9 %) und 7 Patienten (11,5 %) zeigten keine Infiltrate. Mit 39 Patienten (63,9 %) hatte die Mehrheit einen multifokalen Befall aller Lungenlappen. 17 Patienten hatten einen Pleuraerguss (27,9 %). Unter einer mediastinalen Lymphadenopathie litten 28 Patienten (45,9 %) und 6 Patienten waren während der CT-Untersuchung intubiert (6,8 %).

**Table 3:** 

Bildmorph. Eigenschaften	*n*	%/MV ± SD
Typisch	42	68,9 %
Atypisch^a^	5	8,2 %
„Indeterminate“^a^	7	11,5 %
Keine Pneumonie^a^	7	11,5 %
Bilateral	51	83,6 %
Unilateral	3	4,9 %
Pleuraerguss	17	27,9 %
Mediastinale Lymphadenopathie	28	45,9 %
Intubiert	6	6,8 %
*Lappenbefall*		
1 Lappen	3	4,9 %
2 Lappen	5	8,2 %
3 Lappen	5	8,2 %
4 Lappen	2	3,3 %
5 Lappen	39	63,9 %
0 Lappen	7	11,5 %

^a^Als *untypisch* zusammengefasst

### Outcomeparameter

Die Häufigkeiten der Outcomeparameter sind in Tab. 4 zusammengefasst. 16 Patienten hatten einen letalen Verlauf (26,2 %). 27 Patienten benötigten eine intensivmedizinische Behandlung (44,3 %) und 4 Patienten erhielten eine ECMO-Therapie (6,6 %). In unserer Studienpopulation benötigten 13 Patienten (21,3 %) eine nichtinvasive Beatmung/„noninvasive ventilation“ (NIV), 8 Patienten eine High-flow-Sauerstoff-Therapie (13,1 %) und insgesamt 17 Patienten wurden im Verlauf intubiert (27,9 %).

**Table 4:** 

Verlaufsparameter	*n*	%/MV ± SD
Exitus letalis	16	26,2 %
Intensivpflichtigkeit	27	44,3 %
NIV	13	21,3 %
„High flow“	8	13,1 %
Intubiert im Verlauf	17	27,9 %
ECMO-Bedarf	4	6,6 %
*Verlaufs-CT*		
0	48	78,8 %
2	8	13,1 %
3	2	3,3 %
7	1	1,6 %
8	1	1,6 %
16	1	1,6 %

*ECMO* extrakorporale Membranoxygenierung

### Vergleich des Befallsmusters mit Komorbiditäten

Die klinischen Daten und bildmorphologischen Veränderungen für die Patienten mit typischen Infiltraten (Gruppe TYP) und die Patienten mit nichttypischen Befunden (Gruppe UNTYP) ist in Tab. 5 zusammengefasst. In der deskriptiven Statistik konnte keine signifikante Häufigkeit der erfassten Komorbiditäten festgestellt werden. In der Gruppe TYP hatten die untersuchten Patienten etwas häufiger einen Diabetes mellitus Typ 2 (23,8 %) im Gegensatz zur Gruppe UNTYP (15,8 %). Patienten mit einer COPD waren in der Gruppe UNTYP (26,3 %) häufiger vertreten als in der Gruppe TYP (9,5 %).

**Table 5:** 

Parameter	Gruppe TYP *n* = 42		Gruppe UNTYP *n* = 19	
	n	%/MV ± SD	n	%/MV ± SD
Männlich	25	59,5 %	11	57,9 %
Weiblich	17	40,5 %	8	42,1 %
aHTN	24	57,1 %	13	68,4 %
KHK	9	21,4 %	6	31,6 %
HRST	5	11,9 %	1	5,3 %
COPD	4	9,5 %	5	26,3 %
Asthma	4	9,5 %	2	10,5 %
DM II	10	23,8 %	3	15,8 %
PAVK	1	2,4 %	2	10,5 %
Malignom	5	11,9 %	4	21,1 %
Zerebr. Insult	7	16,5 %	2	10,5 %
Normalgewicht	22	52,4 %	11	57,9 %
Übergewicht	10	23,8 %	4	21,1 %
Adipositas I	8	19 %	3	15,8 %
Adipositas II	2	4,8 %	0	0 %
Adipositas III	0	0 %	1	5,3 %
OSAS	2	4,8 %	3	15,8 %
z. N. LAE	7	16,7 %	2	10,5 %
NI	11	26,2 %	4	21,1 %
Reflux	6	14,3 %	2	10,5 %

*MV* „mean value“, *SD* „standard deviation“

### Vergleich bildmorphologische Parameter und Verteilungsmuster

Der Vergleich des Infiltratmusters mit weiteren bildmorphologischen und klinischen Befunden (Tab. 6) zeigte eine signifikante Assoziation des typischen Verteilungsmusters mit einer mediastinalen Lymphadenopathie (*p* = 0,009). Bereits intubierte Patienten mit einem typischen Verteilungsmuster lagen häufiger im Trend (*p* = 0,083) und erhielten im Verlauf signifikant mehr Nachuntersuchungen als Patienten mit nichttypischem Verteilungsmuster (*p* = 0,021). Zusätzlich zeigte sich ein signifikanter Zusammenhang zwischen einem typischen Verteilungsmuster und der bilateralen Manifestation (*p* > 0,001). Ein Pleuraerguss fand sich ohne signifikanten Unterschied in beiden Gruppen.

**Table 6:** 

Parameter	Gruppe TYP *n* = 42		Gruppe UNTYP *n* = 19		*p*-Wert
	n	%/MV ± SD	n	%/MV ± SD	
Mediastinale Lymphadenopathie	24	57,1 %	4	21,1 %	0,009
Intubiert während CT	6	14,3 %	0	0 %	0,083
Verlaufs-CT:	40	–	21	–	0,021
Pleuraerguss	13	31 %	4	21,1 %	0,425
Bilateraler Befall	42	100 %	9	47,4 %	< 0,001
Unilateraler Befall	0	0 %	3	15,8 %	0,008

*MV* „mean value“, *SD* „standard deviation“

### Vergleich Outcomeparameter und Verteilungsmuster

Beim Vergleich des klinischen Verlaufs (Tab. 7) ergab sich eine signifikant erhöhte Mortalität (*p* = 0,012) und eine signifikant höherer Rate an Intensivbehandlungen (*p* < 0,001) bei Patienten mit typischem Infiltratmuster. In der Gruppe TYP wurden Patienten im Verlauf signifikant häufiger intubiert als Patienten in der Gruppe UNTYP (*p* = 0,008). Außerdem erhielten Patienten aus der Gruppe TYP häufiger eine NIV-Behandlung (*p* = 0,040) sowie eine High-flow-Sauerstoff-Therapie (*p* = 0,041). Eine ECMO-Behandlung wurde ausschließlich bei Patienten mit typischem Verteilungsmuster notwendig, aufgrund der geringen Stichprobe allerdings ohne statistisch signifikante Unterschiede (*p* = 0,164).

**Table 7:** 

Parameter	Gruppe TYP *n* = 42		Gruppe UNTYP *n* = 19		*p‑Wert*
	*n*	%/MV ± SD	*n*	%/MV ± SD	
Exitus letalis	15	35,7 %	1	5,3 %	0,012
Intensivpflichtigkeit	25	64,1 %	2	10,5 %	< 0,001
Intubiert im Verlauf	16	38,1 %	1	5,3 %	0,008
NIV	12	28,6 %	1	5,3 %	0,040
„High flow“	8	19,0 %	0	0 %	0,041
ECMO-Bedarf	4	9,5 %	0	0 %	0,164

*MV* „mean value“, *SD* „standard deviation“

*ECMO* extrakorporale Membranoxygenierung, *NIV* nichtinvasive Beatmung/„noninvasive Ventilation“

## Diskussion

Bis dato wurde hauptsächlich der Einfluss von Komorbiditäten auf den Krankheitsverlauf bei COVID-19-Pneumonie demonstriert. Kaeuffer et al. zeigten beispielsweise, dass diverse Risikofaktoren, wie ein hohes Alter, Immunsuppression, Diabetes mellitus, chronische Niereninsuffizienz sowie Adipositas, zu einem deutlich schlechteren Outcome führen [2]. Eine andere Studie aus New York unterstützt diese Aussage [3] und betont, dass das hohe Alter und das männliche Geschlecht deutliche negative Faktoren für das Outcome sind. Untersuchungen, die den Zusammenhang von spezifischen CT-morphologischen Infiltratmustern der COVID-19-Pneumonie mit diesen Risikofaktoren untersucht hat, sind uns bisher nicht bekannt.

### Komorbiditäten und Infiltratmuster

Die typischen Veränderungen im Thorax-CT, die auf eine vorliegende COVID-19-Pneumonie hindeuten könnten, sind peripher betonte bilaterale multifokale Milchglasinfiltrate sowie ein Crazy-paving-Muster [4]. Die Milchglasinfiltrate treten in anderer Verteilung auch bei anderen viralen/aviralen Atemwegsinfektionen, beim Lungenödem oder bei Infarktpneumonien auf [5]. In unserer Analyse waren Patienten mit einer COPD häufiger im Trend für untypische Befunde. In einer Studie aus 2020 resultierte eine bekannte COPD in einer geringeren Mortalität [6]. Passend hierzu zeigten unsere Patienten mit untypischen Befunden ebenfalls eine geringere Mortalität.

Einen statistisch signifikanten Zusammenhang zwischen den untersuchten prädisponierenden Komorbiditäten und einem spezifischen Infiltratmuster konnten wir allerdings nicht nachweisen.

### Outcomeparameter und Infiltratmuster

Patienten mit typischem Infiltratmuster zeigten eine signifikant höhere Mortalität und öfter eine notwendige Intensivbehandlung. Dies ist vereinbar mit den Befunden einer bisherigen Studie, die eine Korrelation der Infiltratgröße mit dem Outcome beobachtete [7]. Eine weitere Studie untersuchte frühe Veränderungen von COVID-19-Patienten im Lungenparenchym mit dem Outcome [8]. Hier wurde ebenfalls gezeigt, dass ein ausgeprägter Lungenbefall mit einem schlechteren Outcome korreliert. Auch Meiler und Kollegen zeigten in ihrer Studie, dass ein „Crazy-paving“-Muster mit pflastersteinartiger Verteilung von pathologischen und normalen Lungenanteilen sowie ein ausgeprägter Lungenbefall ein negatives Outcome prognostizieren können [9]. Keine der Studien verglich allerdings das spezifische Infiltratmuster nach RSNA-Vorlage mit dem klinischen Verlauf.

Anhand unserer Daten ist somit davon auszugehen, dass das Auftreten eines typischen Infiltratmusters die Wahrscheinlichkeit eines schlechten Verlaufs signifikant erhöht. Dies deckt sich auch mit der Beobachtung, dass Patienten mit typischem Infiltratmuster häufiger eine NIV- und High-flow-Therapie benötigten, im Verlauf häufiger intubiert wurden und häufiger Verlaufs-CT erhielten. Eine Studie von Ospina-Tascón et al. zeigte, dass Patienten mit einer COVID-19-Pneumonie von einer nasalen High-flow-Therapie im Vergleich zur konventionellen Sauerstofftherapie klinisch profitieren [10]. Es gibt allerdings auch Studien, die belegen, dass es hinsichtlich der Mortalität der Patienten keinen signifikanten Vorteil einer nasalen High-flow-Therapie gibt wie beispielsweise die Studie von Frat et al. [11] oder Crimi et al. [12].

Die Wirksamkeit der NIV-Therapie bei akuter respiratorischer Insuffizienz war bereits vor der COVID-19-Pandemie bekannt [13]. Zu Beginn der COVID-19-Pandemie wurden die nichtinvasiven Verfahren zur Beatmung frühzeitig eingesetzt. Eine Studie aus Italien zeigte eine verbesserte Prognose bei Patienten, die eine nichtinvasive Beatmung erhielten [14], mit dem Nachteil der erhöhten Infektiosität für das medizinische Personal.

### Bildmorphologie

Bei der Analyse der bildmorphologischen Parameter zeigten Patienten mit typischem Infiltratmuster häufiger eine mediastinale Lymphadenopathie. In vorhergehenden Studien ist diese mit einem schweren Krankheitsverlauf assoziiert [15]. Dies bestätigt wiederum unsere Beobachtung der schlechteren Prognose bei typischem Infiltratmuster.

Die beidseitige Infiltratmanifestation ist ein Kriterium des typischen Infiltratmusters. Die Assoziation bilateraler Infiltrate mit COVID-typischen Befunden ist somit als Korrekturparameter zu werten.

In rezenteren Studien sind bei einigen Patienten mit einer stattgehabten, abgeheilten COVID-19-Pneumonie narbig-retikuläre Veränderungen sowie im Langzeitverlauf auch pulmonale Fibrosen zu finden, wenn in vorherigen CT während der akuten Krankheitsphase Milchglastrübungen zu erkennen waren [16].

### Limitationen

Neben der vergleichsweise geringen Patientenzahl ist das monozentrische Studiendesign als Limitation unserer Studie zu nennen.

## Schlussfolgerung

- Patienten mit typischer COVID-19-Pneumonie bedürfen häufiger einer Intensivbehandlung bzw. weisen eine höhere Mortalität auf als Patienten mit untypischen oder fehlenden pulmonalen Manifestationen in der CT-Bildgebung. Hier lässt sich des Weiteren häufiger eine mediastinale Lymphadenopathie abgrenzen.
- Auch weisen die Patienten mit einem für COVID-19 typischen Infiltratmuster höhere Raten an Intubationen und nichtinvasiven Beatmungsmethoden wie High-flow-Therapie oder NIV auf.
- Ein Zusammenhang zwischen einem spezifischen typischen Infiltratmuster und prädisponierenden Risikofaktoren konnte in unserem Patientenkollektiv nicht nachgewiesen werden, ist aber anzunehmen. Hierzu müssen in einem nächsten Schritt größere Patientenkollektive untersucht werden.
